# Vibration acceleration promotes endochondral formation during fracture healing through cellular chondrogenic differentiation

**DOI:** 10.1371/journal.pone.0229127

**Published:** 2020-03-05

**Authors:** Hiroyuki Yokoi, Yasuhiro Take, Ryohei Uchida, Takuya Magome, Kazunori Shimomura, Tatsuo Mae, Tomoko Okamoto, Tatsuhiro Hanai, Yang Chong, Seira Sato, Minami Hikida, Ken Nakata

**Affiliations:** 1 Medicine for Sports and Performing Arts, Graduate School of Medicine, Osaka University, Osaka, Japan; 2 Department of Orthopedic Surgery, Graduate School of Medicine, Osaka University, Osaka, Japan; 3 Department of Sports Medicine, Yukioka Hospital, Osaka, Japan; Kyungpook National University School of Medicine, REPUBLIC OF KOREA

## Abstract

Vibration acceleration through whole body vibration has been reported to promote fracture healing. However, the mechanism responsible for this effect remains unclear. Purpose of this study was to determine whether vibration acceleration directly affects cells around the fracture site and promotes endochondral ossification. Four-week-old female Wistar Hannover rats were divided into two groups (vibration [V group] and control [C group]). The eighth ribs on both sides were cut vertically using scissors. From postoperative day 3 to 11, vibration acceleration using Power Plate^®^ (30 Hz, low amplitude [30-Low], 10 min/day) was applied in the V group. Mature calluses appeared earlier in the V group than in the C group by histological analysis. The GAG content in the fracture callus on day 6 was significantly higher in the V group than in the C group. The mRNA expressions of SOX-9, aggrecan, and Col-II in the fracture callus on day 6 and Col-X on day 9 were significantly higher in the V group than in the C group. For *in vitro* analysis, four different conditions of vibration acceleration (30 or 50 Hz with low or high amplitude [30-Low, 30-High, 50-Low, and 50-High], 10 min/day) were applied to a prechondrogenic cell (ATDC5) and an undifferentiated cell (C3H10T1/2). There was no significant difference in cell proliferation between the control and any of the four vibration conditions for both cell lines. For both cell lines, alcian blue staining was greater under 30-Low and 50-Low conditions than under control as well as 30-High and 50-High conditions on days 7 and 14. Vibration acceleration under 30-L condition upregulated chondrogenic gene expressions of SOX-9, aggrecan, Col-II, and Col-X. Low-amplitude vibration acceleration can promote endochondral ossification in the fracture healing *in vivo* and chondrogenic differentiation *in vitro*.

## Introduction

The treatment of bone fractures is fundamental and important for the maintenance of physical activity. The clinical outcomes of fracture treatment are generally satisfactory owing to the good healing capacity of bones, as well as improvements in various surgical procedures. However, delayed union and nonunion are still troublesome complications. When they occur, the healing capacity is often impaired, and patients experience a long treatment period or require repeated surgeries, often resulting in disabilities in daily life, with persistent pain and joint stiffness [1]. For the treatment of delayed union and/or nonunion, various approaches have been proposed, including drug therapies, such as administration of vitamin preparations and parathyroid hormone [2–4], and studies have reported that cytokines, such as basic fibroblast growth factor [5,6], and therapies involving mechanical stimuli, such as dynamization for an intramedullary rod [7–11], electromagnetic pulse [12,13], and low-intensity pulsed ultrasound (LIPUS) [14–17], can effectively promote bone fracture healing. However, these approaches have some limitations. Dynamization can only be performed in patients with an intramedullary rod or external fixator. Additionally, although LIPUS is a noninvasive method widely used in clinical practice, its effectiveness is limited in cases involving deep bones and cases in which a metal implant interposes.

In recent years, vibration acceleration using the whole body vibration (WBV) apparatus has been gaining attention as a means for the application of mechanical stimuli to the body. WBV apparatus can provide mechanical loading of alternative gravitational acceleration in the direction and amplitude using three-dimensional vibration for the body, organ, and cells and has been used for acceleration training or exercise. Numerous effects of vibration acceleration have been reported, including improved flexibility and performance, increased blood flow volume and hormonal secretion, muscle hypertrophy, and pain relief [18–24]. Bones, which are sensitive to mechanical stimuli, are also influenced by vibration acceleration, and the approach can certainly improve bone density [25]. In addition, several studies reported that vibration acceleration increased bone formation, mineralization, and bone remodeling in the callus and promoted fracture healing [26–38], and Uchida et al. reported that vibration acceleration with Power Plate^®^ ((Performance Health Systems, LLC, Northbrook, IL)) improved the bone union rate and promoted rib fracture healing on day 12 [39], thereby suggesting that vibration acceleration might be considered a treatment option for delayed union and/or nonunion of bone fractures. As mentioned above, vibration acceleration by WBV has numerous effects, but the mechanism is yet to be fully elucidated.

Considering the fact that vibration acceleration by WBV promotes fracture healing, this study focused on the mechanism of fracture healing promotion in terms of endochondral ossification. Some studies showed enhanced endochondral ossification by WBV [27,30,32]. Wehrle et al. reported that the WBV group, loaded with low-magnitude high-frequency vibration (LMHFV) for 20 min per day, showed the safranin O staining area earlier than the control group in histological evaluation using the mice femoral fracture model [34]. Chung et al. also reported that col-II expression in the WBV group was higher than that in the control group using the osteoporosis rat femoral fracture model [32]. However, other gene expressions related to endochondral ossification and GAG production along with precise molecular mechanisms of vibration acceleration using WBV to chondrocyte progenitor cell itself remain unclear in the cellular level. We hypothesized that vibration acceleration affects chondrogenic cell differentiation during endochondral ossification. To test this hypothesis, we performed experiments using an *in vivo* fracture healing animal model and an *in vitro* chondrogenic differentiation model in cell culture and analyzed gene expression and GAG production.

## Materials and methods

### Commercially available products

For vibration acceleration application *in vivo* and *in vitro*, Power Plate^®^ Pro5HPTM (Performance Health Systems, LLC, Northbrook, IL) was used. This device is able to provide three-dimensional WBV loading in the vertical, horizontal, and sagittal axes under various conditions, with a frequency of 30 or 50 Hz and an amplitude of low amplitude (approximately 2.5 mm) or high amplitude (approximately 5.0 mm). When gravitational acceleration (1 G) was set as the baseline and the direction of gravity was considered positive, the acceleration under 30 Hz-low amplitude (30-L), 30 Hz-high amplitude (30-H), 50 Hz-low amplitude (50-L), and 50 Hz-high amplitude (50-H) conditions periodically reach a peak magnitude of approximately 1.5 G, 3 G, 3 G, and 6 G, respectively.

For biochemical analyses, we used the Blyscan^™^ GAG Assay Kit (Biocolor, Antrim, UK), Alcian Blue 8GX (Sigma-Aldrich, St. Louis, MO), the PureLink RNA mini kit, high-capacity RNA-to-cDNA kit and PureLink genomic DNA kit (Thermo Fisher Scientific, Waltham, MA), and SYBR Green and Taqman Gene Expression Assays (Applied Biosystems, Waltham, MA). For immunohistochemistry, we used the Histofine immunostaining kit (Nichirei, Co., Ltd., Tokyo, Japan) with the anti-SRY-related HMG box-9 (SOX-9) antibody (rabbit polyclonal, D8G8H; Cell Signaling Technology, Danvers, MA). For cell culture experiments, we purchased ATDC5 and C3H10T1/2 cell lines from European Collection of Authenticated Cell Cultures (ECACC; Salisbury, England) and used high-glucose Dulbecco’s Modified Eagle’s Medium (HG-DMEM) and DMEM/F-12 (Wako, Osaka, Japan), fetal bovine serum (FBS) (HyClone; GE Healthcare Life Sciences, Marlborough, MA), penicillin/streptomycin (Gibco BRL, Life Technologies Inc., Carlsbad, CA), ITS+ Premix (BD Biosciences, Bedford, MA), recombinant human BMP-2 (PeproTech, Rocky Hill, NJ), and the premix WST-1 Cell Proliferation Assay System (TAKARA BIO, Shiga, Japan).

### *In vivo* animal experiments

A total of 77 4-week-old female Wistar Hanover rats purchased from Nihon CREA (Tokyo, Japan) were used for the rib fracture model as described previously [39,40]. Briefly, each rat was anesthetized with inhalation of isoflurane and intraperitoneal injection of pentobarbital sodium (5 mg/100 g body weight). A longitudinal incision of 3 cm was made over the thoracic spine, and the superficial back muscles were retracted laterally, thereby exposing the dorsal aspect of the ribs. Then, for rib fracture, the eighth ribs on both sides were cut vertically using a scissor at the lateral margin of the paravertebral muscle. The skin was sutured, and then the animals were maintained in cages, with daily observation of the feeding quantity, body weight, and wound condition. Three rats were housed in each cage (28 × 45 cm and 20-cm height) and were fed a commercial solid diet (CE-2, Nihon CLEA, Tokyo, Japan) and water *ad libitum*. The cages were placed in an animal room in a 12:12 h light:dark cycle, and the temperature and humidity in the animal room were maintained at 20–24°C and 40–60%, respectively.

**Fig 1:**
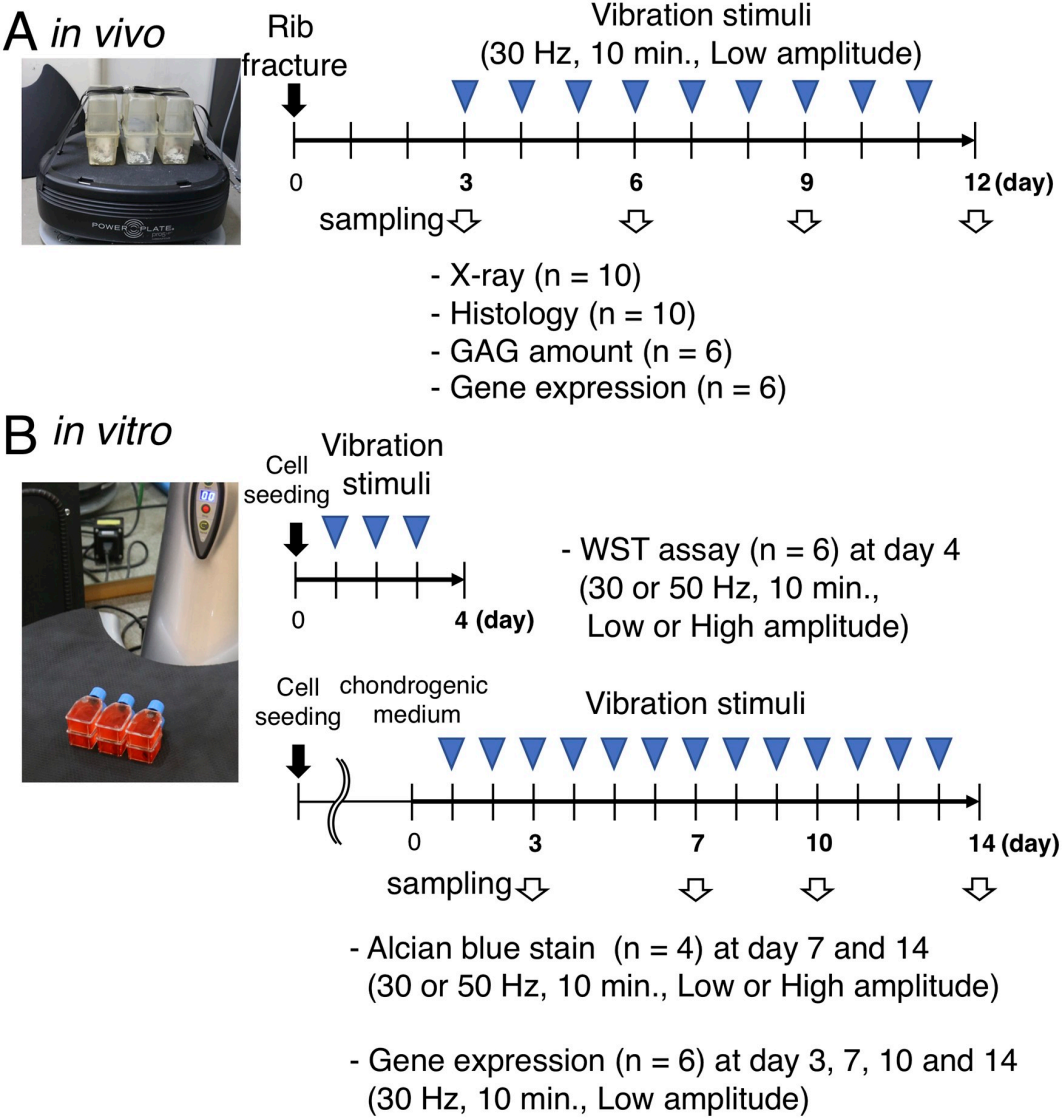
Study design. A: Rats in V-group underwent daily WBV loading for 10 minutes with the frequency of 30-L condition from PFD 3. At PFD 3, 6, 9, and 12, each fracture site was evaluated by X-ray and histological, biochemical and gene expression. B: C3H10T1/2 and ATDC5 cells were used to evaluate the direct cellular effect of vibration stimuli for 10 minutes per day in terms of cell proliferation and differentiation. For cell proliferation, cells were seeded in a T12.5 flask with the normal medium and were exposed to the vibration stimuli of four conditions (30 or 50 Hz, 10 min., Low or High amplitude). At day 4, cell proliferation was evaluated using a WST assay. For chondrogenic differentiation, cells were seeded in a T12.5 flask with the normal medium and expanded to 90% confluency; then the media were changed to the differentiation medium, and vibration stimuli were applied. Chondrogenic differentiation under four conditions (30 or 50 Hz, 10 min., Low or High amplitude) was evaluated to quantify GAG synthesis using alcian blue stain at day 7 and 14 and to analyze gene expressions at day 3, 7, 10, and 14.

The rats were divided into the following two groups: control and vibration groups (C and V groups, respectively). Rats in the V group underwent daily WBV loading for 10 min under the 30-L condition from post fracture day (PFD) 3 until the day before sacrifice (Fig 1A). Each rat was individually placed in a cage, which was secured on Power Plate^®^ using packing tape, to ensure that the animal was exposed to vibration. During vibration acceleration loading, rats in the C group were placed close to Power Plate^®^ to minimize environmental differences, such as noise from Power Plate^®^, between the groups. At PFDs 6, 9, and 12, rats from each group were sacrificed, and whole fractured ribs or 2-mm-thick calluses were harvested for X-ray (10 ribs from each group at each time point), histological (10 ribs from each group at each time point), biochemical (6 calluses from each group at each time point), and gene expression (6 calluses from each group at each time point) analyses as described below. Rats at PFD 3 were sacrificed as preloading controls for these analyses. All experimental procedures were conducted in accordance with the Guide for the Care and Use of Laboratory Animals of the Physiological Society of Japan. The study was also approved by the Animal Use Committee at the Osaka University Graduate School of Medicine (approval ID: 22–071).

#### X-ray and histological evaluation of the fracture

X-rays in two directions (n = 10 from each group at each time point) were obtained to evaluate fracture healing. In the radiological evaluation, the status of healing process was categorized into the following four stages: stage 1 (S1), the gap between the fracture being clear without continuity; stage 2 (S2), the fracture callus being evident but without continuity; stage 3 (S3), the fracture callus bridges the two bones, being evident in one direction of X-ray; and stage 4 (S4), radiographical bone union state with continuous fracture site in two directions of X-rays.

In histological evaluation, the rib specimens (*n* = 10 from each group at each time point) were decalcified with ethylenediaminetetraacetic acid (pH 7.4) after 10% neutral formalin fixation. They were then dehydrated in a graded ethanol series, cut in half along the long axis of the rib, and embedded in paraffin. Sections were prepared (5-μm thick) and were stained with hematoxylin and eosin (HE), Safranin-O, and SOX-9 antibody (immunohistochemical analysis) according to the manufacturer’s instructions. Microscopic images were digitalized using an automated scanning device (Aperio CS2; Leica Biosystems Imaging, Inc., Wetzlar, Germany). The parts of the fracture callus were evaluated with HE and Safranin-O staining according to the previously described four-stage model [41]: inflammation, soft callus formation, hard callus formation, and bone remodeling. Briefly, inflammation begins immediately around the fracture site, where many eosin-stained inflammatory cells exist. In soft callus formation, cartilage tissue appears between the fracture sites, and it is stained with Safranin-O. In hard callus formation, the soft callus area is replaced with new woven bone. In bone remodeling, the hard callus is remodeled with laminar bone [42].

According to the findings of the histological evaluation, we categorized the status of healing maturation into the following four phases: mature 1 (M1) phase, the fracture site was filled with inflammatory tissue; mature 2 (M2) phase, soft callus bridged the fracture site; mature 3 (M3) phase, hard callus became dominant (more than 50% of the callus area in histological sections) in the fracture site; and mature 4 (M4) phase, remodeled bone united with the fractured bone.

#### Biochemical analysis of the fracture callus

The fracture callus (2-mm thick) was harvested from each rib specimen, which was frozen and then crushed using a surgical scissor. The fracture callus specimens were analyzed for GAG content using the Blyscan GAG assay kit according to the manufacturer’s instructions. The GAG content in the fracture callus was normalized to the wet weight of the callus. For gene expression analysis, total RNA was obtained from the fracture callus using the PureLink RNA mini kit, and then, reverse transcription using a high-capacity RNA-to-cDNA kit and real-time RT-PCR using SYBR Green for SOX-9, type II collagen (Col-II), aggrecan (ACN), type X collagen (Col-X), and vascular endothelial growth factor-A (VEGF-A) were performed using sets of specific primers for each gene (Table 1). The relative expression level of each target gene was normalized to that of glyceraldehyde-3-phosphate dehydrogenase (GAPDH).

**Table 1:** Sequences of primers to detect m-RNA level of gene expressions.

Gene	Sequence	Fragment length (bp)	Tm (°C)	
SOX-9	F: 5′-CTGAAGGGCTACGACTGGAC-3′	140	59	Stockl et al.(2013) [43]
	R: 5′-TACTGGTCTGCCAGCTTCCT-3′		60	
ACN	F: 5′-CCCTCACCCCAAGAATCAAGT-3′	178	66	Yu et al. (2012) [44]
	R: 5′-TCATTGGAGCGAAGGTTCTGG-3′		68	
Col-II	F: 5′-AGAACTGGTGGAGCAGCAAGA-3′	124	66	Yu et al. (2012) [44]
	R: 5′-ATCTGGACGTTAGCGGTGTTG-3′		66	
Col-X	F: 5′-CCATGGTTCACACAACCCCTT-3′	129	68	Yu et al. (2012) [44]
	R: 5′-TGGCTGTGGTAAAGCACCTTG-3′		67	
VEGF-A	F: 5′-TGGCTTTACTGCTGTACCTCCA-3′	71	59	Stockl et al. (2013) [43]
	R: 5′-TTTCTGCTCCCCTTCTGTCGT-3′		60	
GAPDH	F: 5′-AACTCCCATTCCTCCACCTT-3′	200	57	Chung et al. (2014) [32]
	R: 5′-GAGGGCCTCTCTCTTGCTCT-3′		57	

### *In vitro* cell culture experiments

C3H10T1/2 (undifferentiated) and ATDC5 (prechondrogenic) cells were used to evaluate the direct cellular effects of vibration acceleration in terms of cell proliferation and differentiation. The C3H10T1/2 and ATDC5 cell lines were maintained in DMEM with 10% FBS and 1% penicillin/streptomycin and DMEM/Ham’s F-12 with 5% FBS and 1% penicillin/streptomycin (normal media), respectively, in a humidified atmosphere of 5% CO_2_ at 37°C. To induce chondrogenic differentiation, recombinant human BMP-2 (300 ng/ml) were added to the C3H10T1/2 culture media and 1% ITS+ Premix were added to the ATDC5 culture media. The culture media were replaced every 2 days. For the application of vibration acceleration in these cells without the risk of contamination or the influence of hydraulic pressure, cells were seeded in a T-12.5 flask in advance with a specific cell number. At the time of loading, the flask was filled with normal medium with the cap closed completely. The flask was secured on Power Plate^®^ using packing tape likewise and was exposed to vibration acceleration for 10 min/day. In this *in vitro* experiment, the four combinations of vibration settings (i.e., 30-L, 30-H, 50-L, and 50-H) were examined. During vibration acceleration loading, flasks of the control group were set outside the incubator to create similar environmental conditions (Fig 1B).

#### Cell proliferation

C3H10T1/2 (3 × 10^4^ cells) or ATDC5 cells (3 × 10^4^ cells) were seeded in a T-12.5 flask with normal medium, and from the following day, they were exposed to vibration acceleration for three days. At days 2, 3, and 4, cell proliferation was assessed using the Premix WST-1 Cell Proliferation Assay System according to the manufacturer’s instructions. After incubation for 2 h at 37°C, the assay medium was analyzed using a spectrometer at 440 nm (*n* = 6 for each vibration condition for each cell line).

#### Chondrogenic differentiation

C3H10T1/2 (5 × 10^5^ cells) or ATDC5 cells (5 × 10^5^ cells) were seeded in a T-12.5 flask with normal medium and were expanded to 90% confluency. Then, the medium was changed to the differentiation medium, and vibration acceleration was applied for designated days (n = 8 in each group). To quantify GAG synthesis, cells were exposed to the four vibration conditions (30-L, 30-H, 50-L, and 50-H), and at days 7 and 14, they were fixed with 10% formalin, washed with 3% acetic acid in distilled water, and stained with Alcian Blue 8GX. After washing, Alcian Blue was extracted with 6-M guanidine–HCl overnight at room temperature, and then the absorbance was measured at 650 nm after temperature equilibration (*n* = 4 from each group). To normalize the absorbance by DNA amount, the DNA amount in both cell lines was measured using the PureLink genomic DNA kit according to the manufacturer’s instructions (*n* = 4 from each group) and averaged. The absorbance of each group normalized by averaged DNA amount was compared.

Chondrogenic gene expressions were examined under the conditions in which chondrogenic differentiation was upregulated by vibration acceleration on 3, 7, 10, and 14 days. After 6 h of the last vibration acceleration, total RNA was extracted as described above, and real-time RT-PCR procedures using TaqMan Gene Expression Assays for *SOX-9*, *Col-II*, *ACN*, *Col-X*, and *VEGF-A* were performed.

### Statistical analysis

The quantitative GAG content and relative expression of each target gene were compared between the two groups at different time points using a two-way analysis of variance (ANOVA), and the absorbance values of the WST-1 cell proliferation assay and Alcian Blue staining were compared among the examined groups using a one-way ANOVA, followed by the Tukey post-hoc test. Analyses were performed using statistical analysis software (PASW Statistics 18.0; SPSS Inc., Chicago, IL, USA). Statistical significance was set at a *p*-value <0.05.

## Results

### Transition of the fracture healing phase according to histology

**Fig 2:**
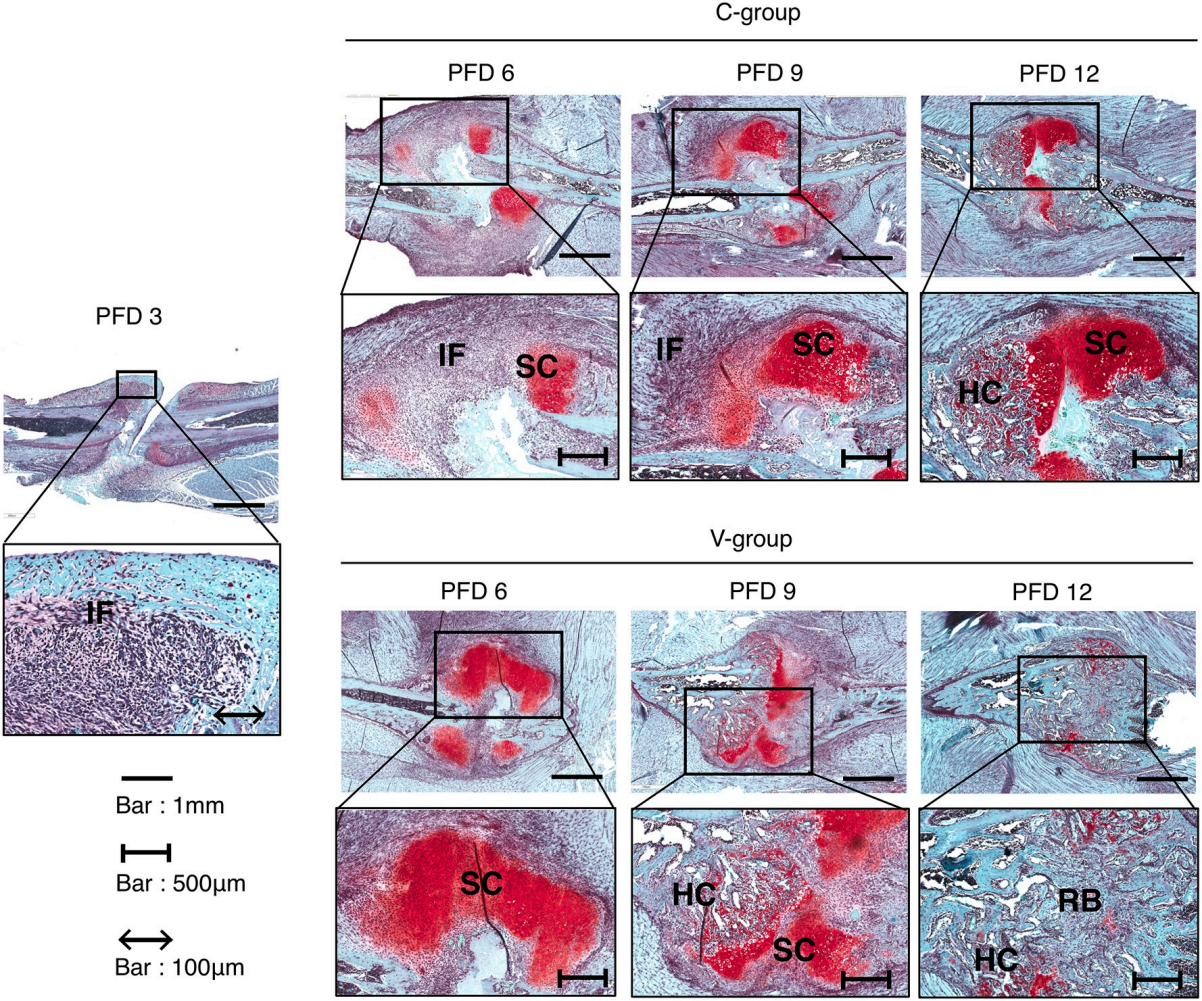
Time course of fracture healing. All images show representative rib fracture sites stained with safranin O in each group. On PFD 3, inflammatory cells infiltrated between the fractured fragments (M1 phase). On PFD 6, the area between fracture fragments was filled with inflammatory cells (M1 phase) in C-group, even though the area of soft callus was dominant at the end and between the fracture fragment (M2 phase) in V-group. On PFD 9, the area of hard callus appeared (M3 phase) in V-group, whereas the area of soft callus remained dominant in C-group (M2 phase). On PFD 12, remodeled bone replaced the hard callus (M4 phase) in V-group, whereas the mixture of soft and hard callus was seen (M3 phase) in C-group. IF: inflammatory area, SC: soft callus, HC: hard callus, RB: remodeling bone.

On PFD 3 (before vibration acceleration), inflammatory cells infiltrated the hematoma between the fractured fragments, and some of these cells formed condensed clusters. Additionally, no positive Safranin-O staining or SOX-9-positive cells were observed. On PFD 6, soft callus characterized by positive Safranin-O staining was observed at the end of the fracture fragments, and the area between fracture fragments was filled with inflammatory cells in the C group, whereas the area of soft callus was dominant at the end of the fracture fragments and between the fracture fragments. On PFD 9, in the V group, the area of soft callus reduced when compared with the finding on PFD 6 and hard callus appeared because of the replacement by soft callus, whereas in the C group, soft callus remained dominant. On PFD 12, in the V group, remodeling bone replaced the hard callus between fracture fragments, whereas in the C group, a mixture of soft callus and hard callus was seen between fracture fragments (Fig 2).

**Fig 3:**
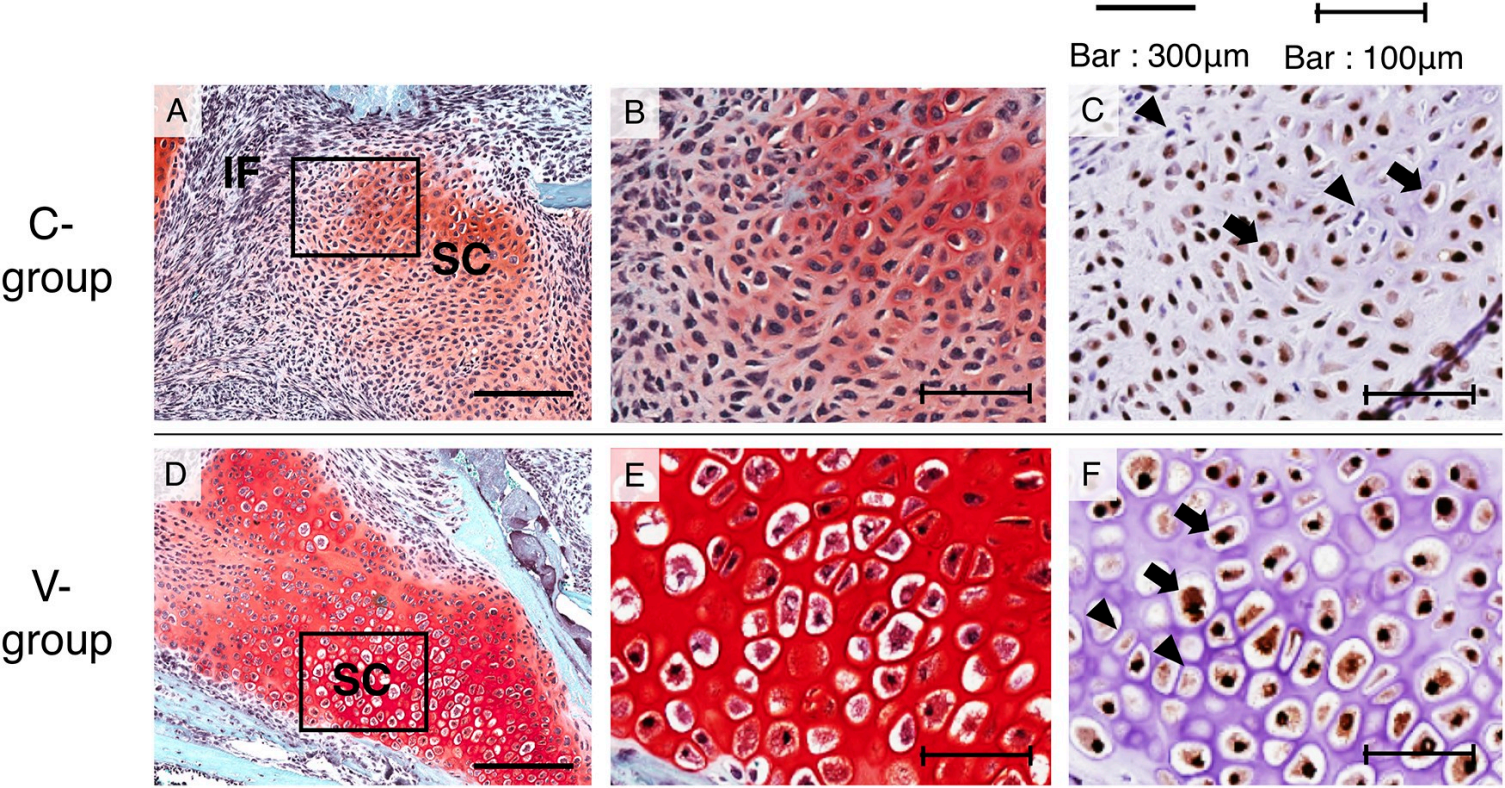
Higher magnification of histological section of callus area on PFD 6. A, B, and C show the callus of C-group; D, E, and F show that of V-group. A, B, D, and E show safranin O-stained images, and C and F show SOX-9 antibody stained images. B, C, E, and F show the squares in images A or D as a high power field image. Black arrow: Sox-9 positive cells, Black triangles Sox-9 negative cells. IF: inflammatory area, SC: soft callus.

On higher magnification of histological sections of the callus area on PFD 6, Sox-9-positive cells were observed in both the C and V groups, although the V group showed hypertrophic chondrocytes and the C group showed abundant proliferative chondrocytes (Fig 3).

**Fig 4:**
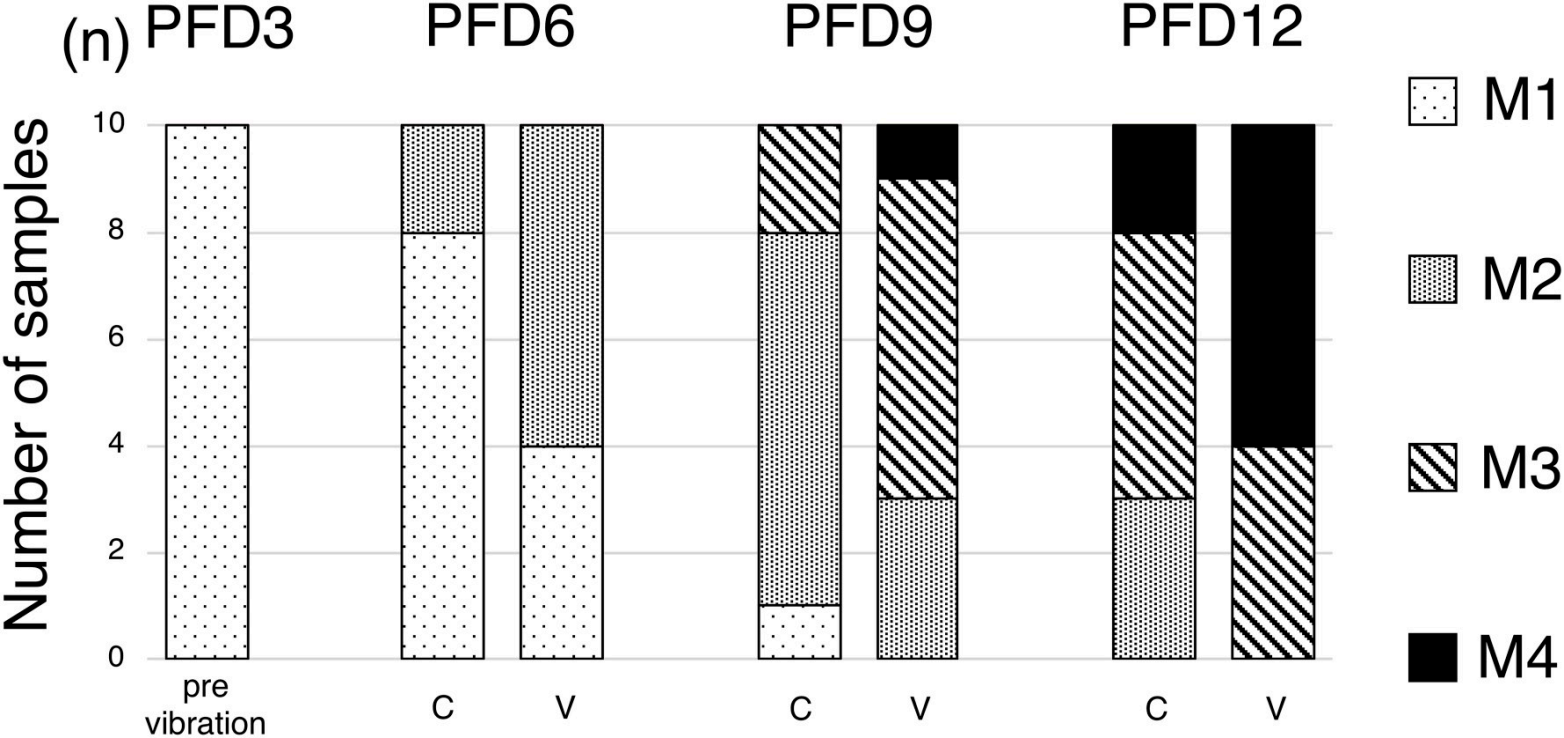
The classification of the status of fracture healing maturation. Maturation phase was graded as M1–M4 phase as described in the text. Maturation phase proceeded earlier in fracture callus in V-group than those in C-group.

With regard to the classification of the status of fracture healing maturation, on PFD 3, all fractured ribs were in the M1 phase. On PFD 6, 2 of the 10 fractured ribs in the C group were in the M2 phase, while 6 of the 10 fractured ribs in the V group were in the M2 phase. On PFD 9, two were in the M3 phase, seven were in the M2 phase, and one was in the M1 phase in the C group, while one was in the M4 phase, six were in the M3 phase, and three were in the M2 phase in the V group. On PFD 12, two were in the M4 phase, five were in the M3 phase, and three were in the M2 phase in the C group, while six were in the M4 phase and four were in the M3 phase in the V group, suggesting that mature calluses appeared earlier in the V group than in the C group (Fig 4).

### X-ray evaluation of the fracture site

**Fig 5:**
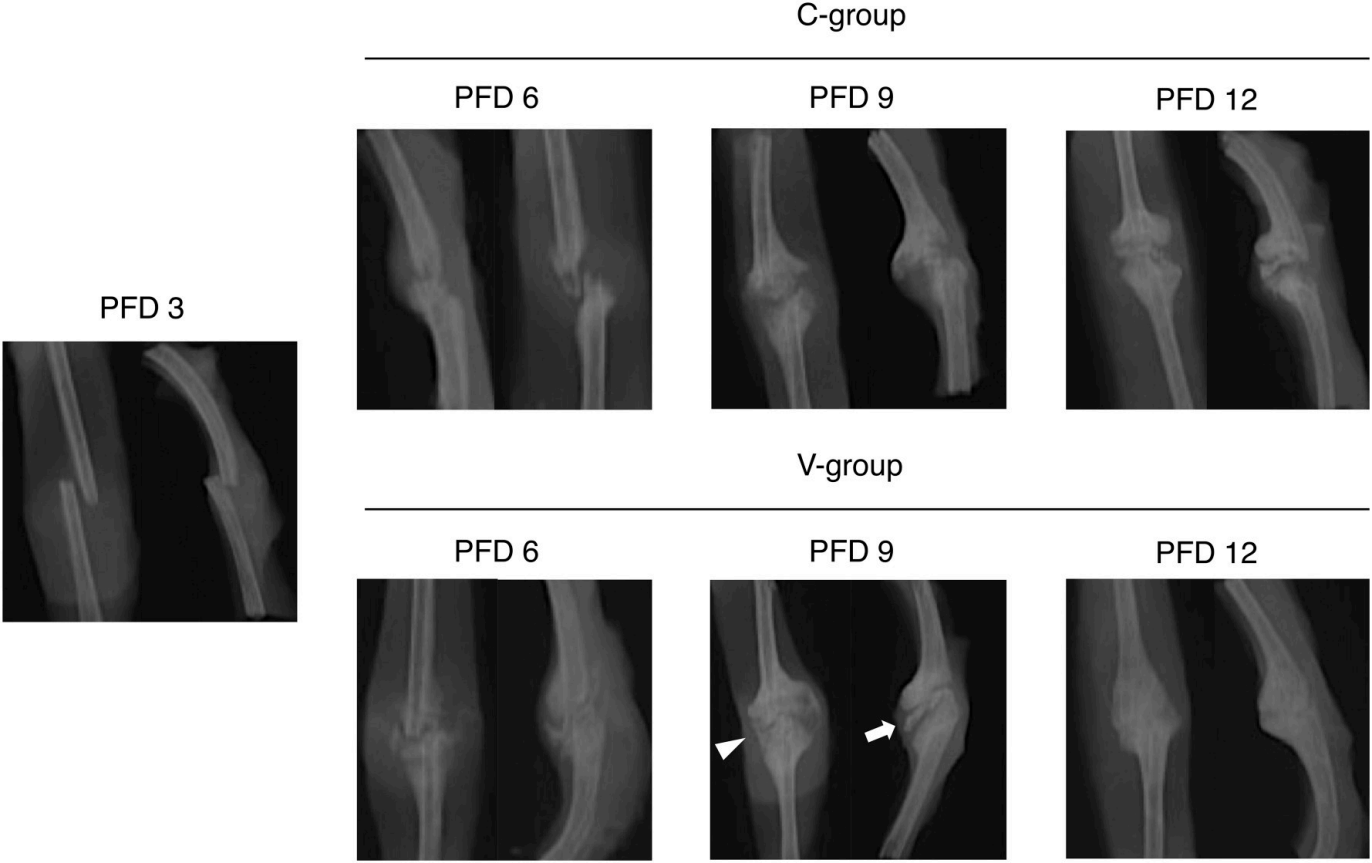
Radiologic time course of fracture healing. All images show representative rib fracture sites in each group. On PFD 3, there was no continuity of the fracture (S1). On PFD 6, a thin calcified image appeared between the fractures (S2) in V group. On PFD 9, the calcification between fractures in V group was thick (white arrow), and bone bridging was observed at least in one direction of X-rays (white triangle) (S3). On PFD 12, the fracture line was unclear in V group with bridging of the fracture in two directions of X-rays (S4).

On PFD 3 (before the vibration acceleration), the gap between the fracture is clear without continuity. All fractures were in S1. In PFD6, fracture callus was evident (S2) in six cases in C-group and in eight cases in V-group, but no callus was observed (S1) in four cases in C group. On PFD 9, the fracture callus bridges two bones at least in one X-ray (S3) direction in eight cases in V-group, but six cases were still in S2 in C-group. On PFD12, five cases in the V-group had radiographical bone union (S4), but only one case in the C-group (Fig 5, Table 2).

**Table 2:** Stage of radiological evaluation in fracture healing.

days	groups	S1	S2	S3	S4
d3		10	0	0	0
d6	C-group	4	6	0	0
	V-group	1	8	1	0
d9	C-group	0	6	4	0
	V-group	0	1	8	1
d12	C-group	0	2	7	1
	V-group	0	0	5	5

### GAG content in the fracture callus

Callus wet weights on PFDs 3, 6, 9, and 12 were not significantly different between the C and V groups. In the C group, the GAG content of the callus increased from PFD 3 and peaked on PFD 9 (Table 3). In the V group, the GAG content of the callus peaked on day 6, and at this point, it was significantly higher in the V group than in the C group.

**Table 3:** GAG contents in the fracture callus.

Groups	PFD 3	PFD 6	PFD 9	PFD 12
**C group (ng/wet weight(mg))**	173.1 ± 19.0	234.6 ± 10.1 ^a^	295.8 ± 46.5	270.7 ±22.8
**V group (ng/wet weight(mg))**		332.6 ± 21.8 ^a^	311.6 ± 48.0	256.3 ±29.1

^a^: p<0.05

### Quantitative gene expression analysis in the fracture callus

**Fig 6:**
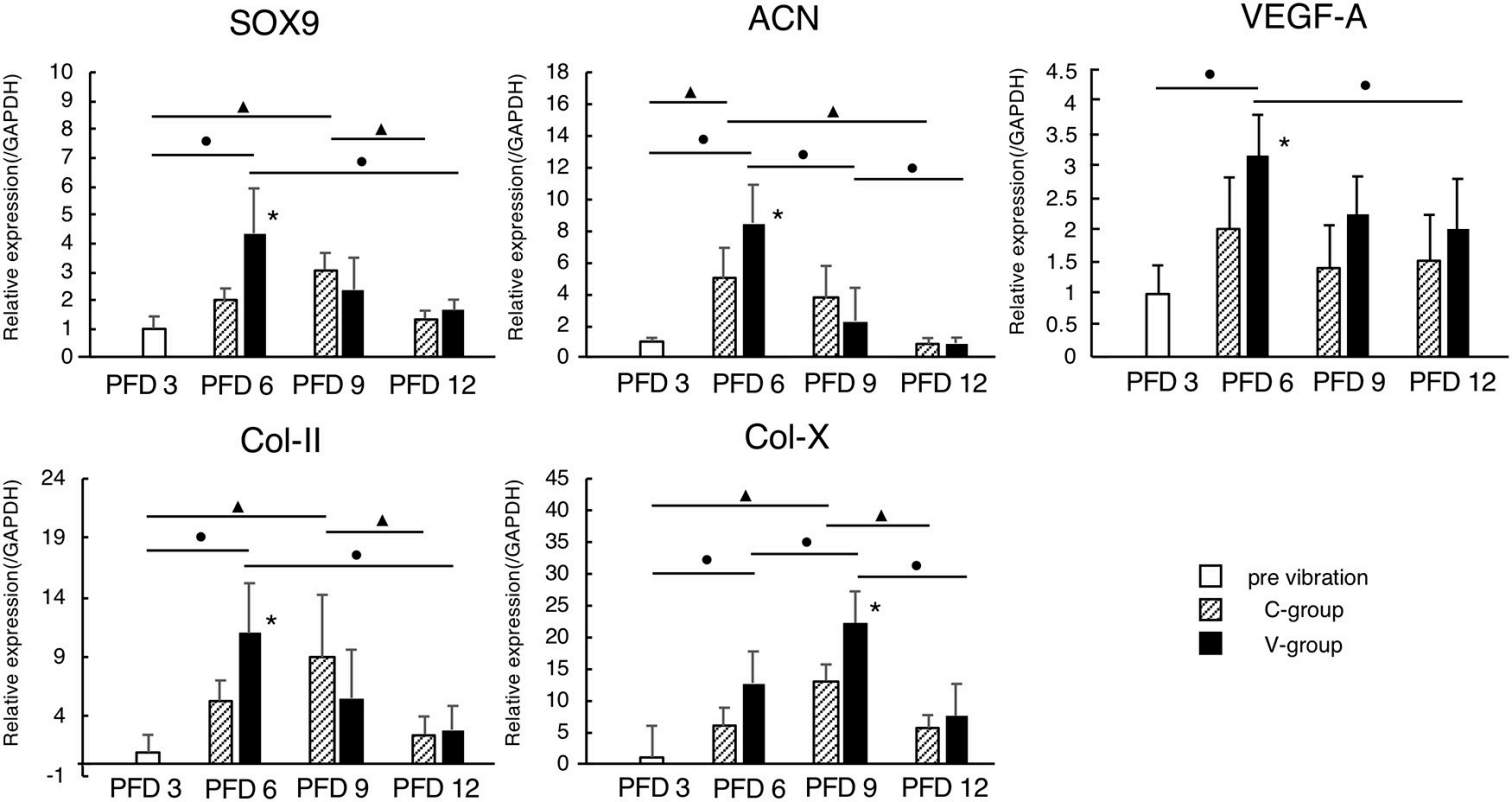
Quantitative gene expression analysis in the fracture callus. In the C-group, the mRNA expression levels of SOX-9 and Col-II in the fracture callus increased from PFD 3 and peaked on PFD 9, whereas in the V-group, these levels peaked on PFD 6, and at this point, the levels were significantly higher in the V-group than those in the C-group. Additionally, the mRNA expression levels of ACN and Col-X increased from PFD 3, the level of ACN peaked on PFD 6, and the level of Col-X peaked on PFD 9 in both groups. However, the peak levels of ACN and Col-X were significantly higher in the V-group than those in the C-group. Moreover, the mRNA expression level of VEGF-A increased from PFD 3 and peaked on PFD 6 in both groups, and the peak level was significantly higher in the-V group than in the C-group. *: p < 0.05. The black circle indicates a significant difference in the V-groups, and the black triangle in the C-group.

In the C group, the mRNA expression levels of *SOX-9* and *Col-II* in the fracture callus increased from PFD 3 and peaked on PFD 9, whereas in the V group, these levels peaked on PFD 6, and at this point, the levels were significantly higher in the V group than those in the C group (Fig 6). Additionally, the mRNA expression levels of *ACN* and *Col-X* increased from PFD 3, the level of *ACN* peaked on PFD 6, and the level of *Col-X* peaked on PFD 9 in both groups. However, the peak levels of *ACN* and *Col-X* were significantly higher in the V group than those in the C group. Moreover, the mRNA expression level of *VEGF-A* increased from PFD 3 and peaked on PFD 6 in both groups, and the peak level was significantly higher in the V group than in the C group (Fig 6).

### *In vitro* cell proliferation and GAG production

Cell proliferation from day 2 to day 4 was observed under any conditions for either the ATDC5 or C3H10T1/2 cell line. In each group the number of cells increased from day 2 to day 4, however there was no significant difference between the control and any of the four vibration conditions at days 2, 3, and 4 (Table 4).

**Table 4:** Cell proliferation of ATDC5 or C3H10T1/2 cell line.

		C	30-L	30-H	50-L	50-H
ATDC5	d2	0.52 ± 0.12 ^a^^,^ ^b^	0.6 ± 0.21 ^d^	0.41 ± 0.14 ^f^^,^ ^g^	0.7 ± 0.26 ^i^	0.51 ± 0.17 ^j^
	d3	0.85 ± 0.21 ^a^^,^ ^c^	1.01 ± 0.42 ^e^	0.94 ± 0.32 ^f^^,^ ^h^	1.12 ± 0.16	0.89 ± 0.31 ^k^
	d4	1.23 ± 0.34 ^b^^,^ ^c^	1.31 ± 0.52 ^d^^,^ ^e^	1.55 ± 0.38 ^g^^,^ ^h^	1.47 ± 0.34 ^i^	1.20 ± 0.35 ^j^^,^ ^k^
C3H10T1/2	d2	0.31 ± 0.06 ^l^^,^ ^m^	0.36 ± 0.06 ^o^	0.29 ± 0.09 ^q^	0.34 ± 0.05 ^s^^,^ ^t^	0.33 ± 0.09 ^v^
	d3	0.52 ± 0.08 ^l^^,^ ^n^	0.55 ± 0.05 ^p^	0.49 ± 0.04 ^r^	0.55 ± 0.15 ^s^^,^ ^u^	0.45 ± 0.04 ^w^
	d4	0.80 ± 0.06^m^^,^ ^n^	0.81 ± 0.10 ^o^^,^ ^p^	0.70 ± 0.11 ^q^^,^ ^r^	0.78 ± 0.10 ^t^^,^ ^u^	0.67 ± 0.09 ^v^^,^ ^w^

^a, b, c, d, e, f, g, h, i, j, k, l, m, n, o, p, q, r, s, t, u, v^ and ^w^: p < 0.05

**Fig 7:**
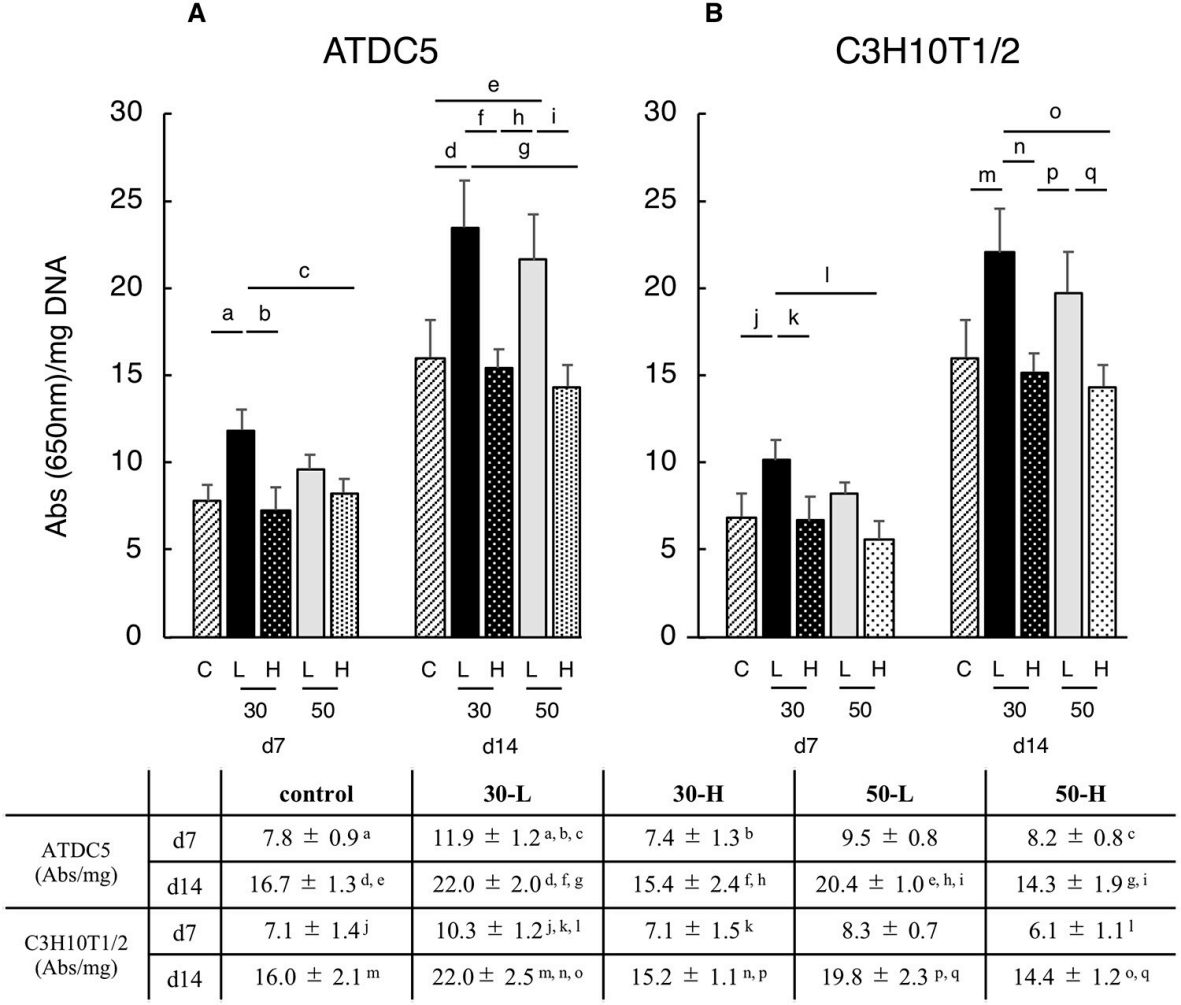
GAG production of ATDC5 and C3H10T1/2 cell lines. ATDC5 and C3H10T1/2 cells showed significantly higher absorbance values of Alcian Blue staining under the 30-L condition than the control, 30-H and 50-H conditions on day 7. Furthermore, both cell lines showed significantly higher absorbance values under the 30-L and 50-L conditions than those under control, 30-H and 50-H conditions on day 14 (except for the C3H10T1/2 cell line between control and 50-L conditions). a, b, c, d, e, f, g, h, i, j, k, l, m, n, o, p and q: p < 0.05.

There was no significant difference between the control and any of the four vibration conditions at days 7 and 14 in averaged DNA amount of each conditions (Table 5). When chondrogenic differentiation was induced, ATDC5 and C3H10T1/2 cells showed significantly higher absorbance values of Alcian Blue staining under the 30-L condition than the control, 30-H and 50-H conditions on day 7. Furthermore, both cell lines showed significantly higher absorbance values under the 30-L and 50-L conditions than those under control, 30-H and 50-H conditions on day 14 (except for the C3H10T1/2 cell line between control and 50-L conditions) (Fig 7).

**Table 5:** DNA amount of ATDC5 or C3H10T1/2 cell line.

DNA amount		C	30-L	30-H	50-L	50-H
ATDC5 (μg)	d7	28.4 ± 2.6	22.5 ± 1.6	26.1 ± 2.6	24.4 ± 2.9	22.1 ± 2.6
	d14	18.3 ± 2.8	20.7 ± 4.2	18.8 ± 1.3	19.5 ± 1.0	17.7 ± 3.0
C3H10T1/2 (μg)	d7	27.7 ± 3.4	26.5 ± 3.3	28.8 ± 3.9	28.7 ± 4.9	28.8 ± 3.7
	d14	18.3± 2.9	20.8 ± 4.2	18.9 ± 1.4	20.5 ± 2.0	17.7± 3.0

### Quantitative analysis of gene expression in ATDC5 and C3H10T1/2 cell lines

**Fig 8:**
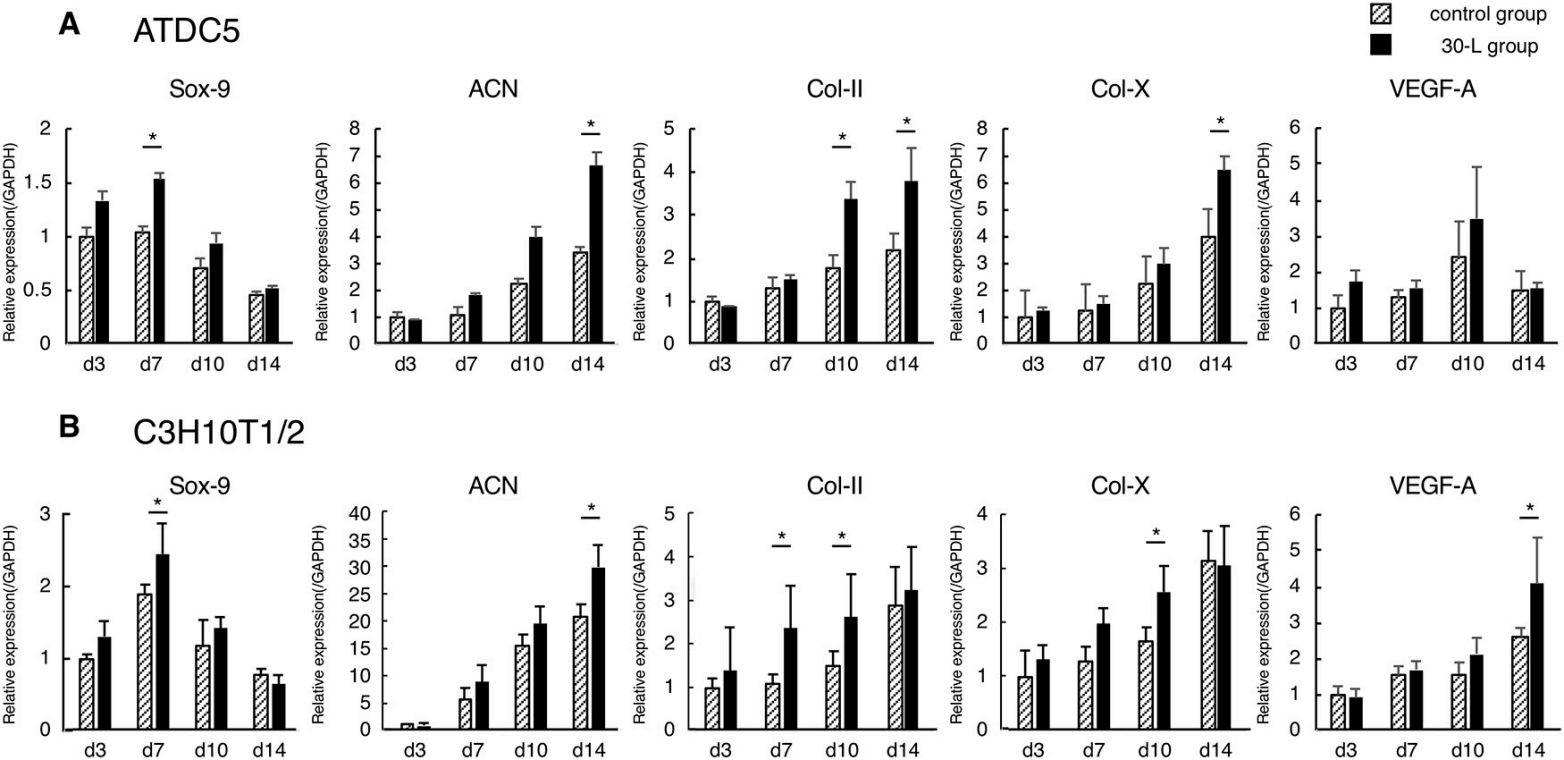
Quantitative analysis of gene expression in ATDC5 and C3H10T1/2 cell lines. The mRNA expressions of SOX-9 on day 7, Col-II on days 10 and 14, and ACN on day 14 in the ATDC5 cell line and SOX-9 on day 7, Col-II on days 7 and 10, and ACN on day 14 in the C3H10T1/2 cell line were significantly higher in the V-group than those in the C-group. The mRNA expressions of Col-X in both cell lines and the expression of VEGF-A in the C3H10T1/2 cell line were significantly higher in the V group than those in the C group on day 14. *: p < 0.05.

Vibration acceleration under the 30-L condition upregulated the expressions of several chondrogenic genes in both cell lines. The mRNA expressions of SOX-9 on day 7, Col-II on days 10 and 14, and ACN on day 14 in the ATDC5 cell line and SOX-9 on day 7, Col-II on days 7 and 10, and ACN on day 14 in the C3H10T1/2 cell line were significantly higher in the V group than those in the C group (Fig 8). Furthermore, the mRNA expressions of Col-X in both cell lines and the expression of VEGF-A in the C3H10T1/2 cell line were significantly higher in the V group than those in the C group on day 14.

## Discussion

It is widely known that mechanical stimulation enhances fracture healing [7–17]. To elucidate the mechanism responsible for improved fracture healing, we first performed a histological time-course analysis using the same rib fracture model as the previous study by Uchida et al. from day 3 to day 12, which suggested that fracture healing under vibration acceleration follows the normal process involving callus formation and bone remodeling at the fracture site through an inflammation stage, but mature calluses appeared earlier in the V group than in the C group. This was confirmed by higher GAG content in the callus at the fracture site on day 7 as well as upregulation of chondrogenic gene expression in the vibration-stimulated callus at the immature phase of PFDs 6–9. On biochemical analysis of the entire callus, both the GAG content and gene expressions reached their peak values at an earlier point in the V group than in the C group. These *in vivo* results indicated that the effects of vibration acceleration on callus maturation were exerted at the early phase of fracture healing, as stimulation was applied from PFD 3, when the fracture site was filled with inflammatory tissue, i.e., the M1 phase, and the V and C groups differed on PFD 6 with regard to the appearance of soft callus bridging at the fracture site. Accelerated soft callus formation in the V group subsequently potentiated endochondral ossification of the callus to form hard callus, and this resulted in accelerated fracture healing. To further elucidate the direct target of vibration acceleration, we analyzed cellular responses against vibration in *in vitro* cultures of the prechondrogenic cell line ATDC5 and undifferentiated cell line C3H10T1/2. In both the ATDC5 and C3H10T1/2 cell lines, GAG synthesis was high not only among cells stimulated with 30-L vibration (similar to the *in vivo* experiment), but also among those stimulated with 50-L vibration. Moreover, chondrogenic gene expressions were upregulated by vibration acceleration in the cell lines. Considering the *in vivo* and *in vitro* results together, we believe that vibration acceleration could promote chondrogenic differentiation in the fracture callus, at least at the cellular level, which eventually accelerates fracture healing. Fracture healing *in vivo* may be affected by the alteration of blood flow and/or the hormonal effect of vibration acceleration [23,24].

Previously, several studies reported accelerated fracture healing *in vivo* with low-magnitude high-frequency vibration (LMHFV) stimuli and concurrently observed alterations in morphology and gene expressions, such as an increased cartilaginous callus area on histological assessment [27], upregulated *Col-II* mRNA expression [32], and increased angiogenesis and upregulated *VEGF* expression [30]. The former two studies suggested that LMHFV could enhance chondrogenesis in the callus, and the findings in the latter study might be explained by accelerated terminal differentiation of chondrocytes, which eventually triggers VEGF expression and induces angiogenesis. Our *in vivo* results demonstrated not only the accelerated induction of the M2 phase and the upregulation of Col-II and VEGF expression levels, which are consistent with the findings in previous studies [30,32], but also high GAG content and upregulation of the expressions of other chondrogenic genes in the fracture callus, strongly indicating that vibration acceleration under the 30-L condition enhances the endochondral ossification process during fracture healing.

Our *in vitro* results indicated the direct effects of vibration acceleration on local chondroprogenitors. Mechanical stress on bone and cartilage in the living body plays a physiologically important role. Several groups reported that osteocytes have mechanoreceptors, and optimal mechanical stress for osteocytes promotes bone metabolism [45,46]. Additionally, LIPUS directly acts on mesenchymal stem cells, osteoblasts, chondrocytes, or osteoclasts and affects their proliferation or differentiation [17]. In several studies published recently, vibration acceleration has been reported to promote osteogenic or adipogenic differentiation from bone marrow stromal cells [47–49]. Furthermore, Marycz et al. reported that vibration acceleration under the conditions of 0.3 G, 35 Hz, for 15 minutes per day enhanced the chondrogenic differentiation of human adipose-derived mesenchymal stromal stem cells [50]. The findings of these studies suggest that vibration acceleration may be directly effective for various kinds of cell differentiations. Despite a difference in the peak-to-peak magnitude between these previous studies (0.3 G) and our study, we also found that vibration acceleration under 30-L and 50-L conditions directly affected the prechondrogenic cell line and triggered high production of GAG as well as upregulation of chondrogenic gene expressions, while vibration acceleration under 30-H and 50-H conditions was ineffective. Unlike previous studies that used a custom-made vibration apparatus, we consistently used Power Plate^®^, which is a commercially available product for use in humans, in the *in vivo* and *in vitro* experiments in consideration of its future clinical application. Our *in vivo* and *in vitro* results collectively suggested that local prechondrogenic cells themselves respond to vibration acceleration and that vibration acceleration under certain conditions could promote chondrogenic differentiation, resulting in accelerated soft callus formation and consequent accelerated fracture healing.

As described above, the conditional difference with regard to vibration acceleration for the promotion of chondrogenic differentiation was interesting. Both C3H10T1/2 and ATDC5 cell lines demonstrated significantly higher absorbance for alcian blue staining under 30-L and 50-L conditions than under control as well as 30-H and 50-H conditions. When these vibration conditions were converted to gravitation acceleration, both 50-L and 30-H conditions had a peak-to-peak magnitude of approximately 3.0 G, although the former was effective and the latter was not in terms of increased alcian blue staining. The 30-L condition was also effective in this context; however, its magnitude was calculated as approximately 1.5 G. These results suggest that enhanced chondrogenic differentiation may be determined by the amplitude of the vibration condition, which should be low, and may not be determined by the frequency or gravitational acceleration magnitude, at least when Power Plate^®^ is used to apply vibration acceleration. Uchida et al. reported the *in vivo* results of Power Plate^®^ and mentioned that an improved bone union rate and large heterotopic ossification were obtained when vibration acceleration under a low amplitude condition was applied (same as the 30-L condition in our study), whereas vibration acceleration under a high amplitude condition (30-H) was not effective [39]. It is somewhat striking that their findings and our findings were concurrently in favor of the 30-L condition, regardless of the differences between the assessed animal species (i.e., rat and mouse) and the differences between *in vivo* and *in vitro* experiments. Taken together, the promotive effect on fracture healing may be dependent on the condition of WBV, and for Power Plate^®^, the amplitude setting may be important. With regard to other devices of WBV, a systematic review on their effects on fracture healing has recently been published and has presented controversial results among *in vivo* studies [51]. These differences might be attributable to differences in the animal models and animal species as well as differences in the employed vibration devices and conditions. Historically, LMHFV, which is not defined strictly but is generally referred to as a vibration condition with a peak-to-peak magnitude of 0.3 G and an amplitude less than 0.1 mm, was regarded as a favorable condition in many of these studies [26–30,32,37,38,50,52]. However, as our results suggested that magnitude might not be an important factor, one should not be too adherent to LMHFV. Rather, for the purpose of clinical application, consistent use of the same vibration apparatus and exploratory investigations for an optimal condition throughout a bench-to-bedside process may be mandatory. In this context, we have provided positive *in vivo* data and have presented the associated mechanism through *in vitro* experiments, which may help in the future clinical application of Power Plate^®^.

The present study has some limitations. First, the vibration magnitude, frequency, and duration were limited. These stimulation conditions might influence the biological effects, and optimal conditions for bone healing might be obtained through the modulation of variables. Second, this study evaluated endochondral ossification in the fracture healing process through both histological and biochemical analyses, whereas intramembranous ossification was not evaluated in detail. Both endochondral ossification and intramembranous ossification are important in the healing process. *In vitro* studies mentioned that the osteogenic differentiation and ossification of the MC3T3-E1 cell line or mesenchymal stem cells were promoted by several mechanical stresses [47,53–56]. Although we did not detect the apparent difference in intramembranous ossification through histological analysis, intramembranous ossification might be promoted by vibration acceleration. This remains to be elucidated in detail in the future. Finally, the effects of vibration acceleration were not thoroughly evaluated, except for the influence on the cell itself. Various effects of vibration acceleration, such as changes in blood flow volume, hormonal secretion (growth hormones, sex hormones, and insulin-like growth factors), and bone metabolism, have been reported [18–22,57–64]. These effects might influence each other with regard to fracture healing *in vivo*, and further studies are needed. Nevertheless, we have shown that vibration acceleration has a direct effect on chondrogenic differentiation at least at the cellular level.

## Conclusion

Vibration acceleration at low amplitude and 30-Hz frequency using Power Plate^®^ can promote endochondral callus ossification and thus promote fracture healing. Additionally, Vibration acceleration at low amplitude of 30-Hz and 50-Hz can promote chondrogenic differentiation *in vitro* in ATDC5 and C3H10T1/2.
